# Stability of vancomycin hydrochloride employed in antimicrobial seal solutions of central intravenous catheters

**DOI:** 10.1590/1518-8345.5869.3621

**Published:** 2022-08-01

**Authors:** Daniele Porto Barros, Priscilla Sete de Carvalho Onofre, Fernando Luiz Affonso Fonseca, Paulo César Pires Rosa, Mavilde da Luz Gonçalves Pedreira, Maria Angélica Sorgini Peterlini

**Affiliations:** 1 Universidade Federal de São Paulo, Escola Paulista de Enfermagem, São Paulo, SP, Brasil.; 2 Bolsista da Coordenação de Aperfeiçoamento de Pessoal de Nível Superior (CAPES), Brasil.; 3 Universidade Estadual de Campinas, Faculdade de Ciências Farmacêuticas, Campinas, SP, Brasil.; 4 Universidade Federal de São Paulo, Instituto de Ciências Ambientais, Químicas e Farmacêuticas, Diadema, SP, Brasil.

**Keywords:** Catheter-Related Infections, Drug Stability, Infusions, Intravenous, Vancomycin, Heparin, Nursing, Infecções Relacionadas a Cateter, Estabilidade de Medicamentos, Infusões Intravenosas, Vancomicina, Heparina, Enfermagem, Infecciones Relacionadas con Catéteres, Estabilidad de Medicamentos, Infusiones Intravenosas, Vancomicina, Heparina, Enfermería

## Abstract

**Objective:**

to verify the stability of vancomycin hydrochloride in antimicrobial seal solutions with and without association of heparin sodium according to temperature and association time.

**Method:**

an experimental study designed for the analysis of hydrogenionic potential and concentration by means of high-efficiency liquid chromatography of vancomycin hydrochloride (n=06) and vancomycin hydrochloride and heparin sodium (n=06). The solutions studied were submitted to absence of light, as well as to 22°C and 37°C. Analyses in triplicate (n=192) were performed at the initial moment (T0) and three (T3), eight (T8) and 24 hours (T24) after preparation. The data were submitted to analysis of variance (p≤0.05).

**Results:**

concentration of the antimicrobial at 22°C presented a reduction (T0-T8) and a subsequent increase (T24); hydrogenionic potential decreased significantly over time. At 37°C, the concentration increased up to T3 and decreased at T24, with a reduction of hydrogenionic potential up to 24 hours. Concentration of the vancomycin hydrochloride and heparin sodium solutions varied with a reduction at 22°C, accompanied by increased hydrogenionic potential. Precipitate formation was observed by visual inspection of the vancomycin hydrochloride-heparin sodium association (T3).

**Conclusion:**

pharmacological stability of vancomycin hydrochloride (5 mg/mL) and physical incompatibility with heparin sodium (100 IU/mL) were evidenced after three hours of association in the antimicrobial seal solutions studied.

## Resumo

**Objetivo:**

verificar a estabilidade do cloridrato de vancomicina em soluções de selo antimicrobiano sem e com associação de heparina sódica segundo a temperatura e tempo de associação.

**Método:**

estudo experimental delineado para análise de potencial hidrogeniônico e concentração por cromatografia líquida de alta eficiência de soluções de cloridrato de vancomicina (n=06) e cloridrato de vancomicina e heparina sódica (n=06). Submeteram-se as soluções estudadas à ausência de luz, 22°C e 37°C. Análises em triplicadas (n=192) ocorreram no momento inicial (T0), três (T3), oito (T8) e 24 horas (T24) após preparo. Os dados foram submetidos à análise de variância (p≤0,05).

**Resultados:**

a concentração do antimicrobiano a 22°C apresentou redução (T0-T8) e posterior elevação (T24); o potencial hidrogeniônico diminuiu significativamente ao longo do tempo. Em 37°C a concentração aumentou em até T3 e reduziu em T24, com redução de potencial hidrogeniônico até 24 horas. A concentração das soluções de cloridrato de vancomicina e heparina sódica apresentaram variação com redução a 22°C acompanhada de aumento de potencial hidrogeniônico. Observou-se formação de precipitado por inspeção visual da associação cloridrato de vancomicina e heparina sódica (T3).

**Conclusão:**

evidenciou-se estabilidade farmacológica do cloridrato de vancomicina (5 mg/mL) e incompatibilidade física com heparina sódica (100 UI/mL) após três horas de associação nas soluções de selo antimicrobiano estudadas.

## Resumen

**Objetivo:**

verificar la estabilidad del clorhidrato de vancomicina en soluciones de sellado antimicrobiano solo y combinado con heparina sódica según la temperatura y el tiempo de combinación.

**Método:**

estudio experimental diseñado para analizar el potencial de hidrógeno y la concentración por cromatografía líquida de alta resolución de soluciones de clorhidrato de vancomicina (n=06) y de clorhidrato de vancomicina y heparina sódica (n=06). Las soluciones estudiadas fueron sometidas a ausencia de luz, 22°C y 37°C. Se realizaron análisis por triplicado (n=192) en el momento inicial (T0), a las tres (T3), ocho (T8) y 24 horas (T24) después de la preparación. Los datos fueron sometidos a análisis de varianza (p≤0,05).

**Resultados:**

la concentración de antimicrobiano a 22°C mostró una reducción (T0-T8) y un posterior aumento (T24); el potencial de hidrógeno disminuyó significativamente con el tiempo. A 37°C, la concentración aumentó hasta T3 y disminuyó en T24, el potencial de hidrógeno disminuyó hasta las 24 horas. La concentración de las soluciones de clorhidrato de vancomicina y heparina sódica mostró variación con la reducción a 22°C acompañada de un aumento del potencial de hidrógeno. Mediante inspección visual se observó la formación de un precipitado al combinar clorhidrato de vancomicina y heparina sódica (T3).

**Conclusión:**

el clorhidrato de vancomicina (5 mg/ml) presentó evidencia de estabilidad farmacológica e incompatibilidad física con la heparina sódica (100 UI/ml) después de las tres horas de haberse realizado la combinación en las soluciones de sellado antimicrobiano estudiadas.

**Highlights**

(1) Both temperatures led to a change in pH and concentration of the antimicrobial.

(2) Vancomycin hydrochloride at 22°C obtained less variation in chemical behavior.

(3) Association of the drugs resulted in a change in physical stability.

(4) Association of the drugs resulted in a solution with physical incompatibility.

## Introduction

Critically-ill patients often require multiple drugs, almost all of them intravenously, in order to guarantee the plasma levels in the concentration and time required for an adequate therapeutic response^1^. Consequently, the use of central intravenous catheters (CICs) has been indicated, due to the characteristics of most of the drugs employed, osmolarity and hydrogenionic potential (pH), among others^2^ ^-^ ^4^. 

CIC preservation in patients with chronic diseases is essential. However, prolongation of the use time of these devices is a predisposing factor for central catheter-related bloodstream infection (CCRBSI)^5^ ^-^ ^6^, due to biofilm formation in the lumen of the catheter^7^. The traditional intervention involves removal of the device. However, this is not always feasible for these patients due to the restricted vascular network resulting from multiple punctures and treatment, as well as from the clinical condition, factors that hinder invasive interventions or procedures. 

Thus, use of the seal technique with antimicrobials in CICs emerges as an adjuvant in the treatment^2^ ^,^ ^5^. It comprises the administration of antimicrobials with a strong recommendation for the use of vancomycin hydrochloride^5^ ^,^ ^8^ ^-^ ^9^ in the lumen of the catheter at a concentration 100 to 1,000 times higher than the minimum inhibitory value usually employed for systemic therapy^8^, being frequently used in combination with heparin sodium^10^ ^-^ ^11^. 

The solution used in the lumen of the CIC has the function of decontamination, being a *sine qua non* condition for maintaining stability of the drugs within the device during the period indicated for the expected effect^8^ ^,^ ^11^. 

A number of studies indicate that the antimicrobial seal may remain in the lumen of the intravenous catheter for a long period of time, in order to overcome the barrier formed by the microbial biofilm^8^ ^-^ ^9^ ^,^ ^11^ ^-^ ^12^. 

However, there is no evidence related to the maximum volume to be administered, permanence time of the solution in the catheter lumen, use frequency and adequate concentration, as well as the association with an anticoagulant^5^ ^,^ ^8^ ^,^ ^12^. Most clinical studies propose a minimum of eight hours a day to achieve the expected action^8^ ^-^ ^9^ ^,^ ^12^. However, there is also the recommendation for the solution to remain between 24 and 48 hours inside the CIC^8^. 

Thus, the concern about antimicrobial stability in association with heparin sodium remains. Although these phenomena are dependent, among other factors, on concentration of the drugs, exposure to higher temperatures and contact time between the medications should also be evaluated^9^ ^,^ ^11^ ^-^ ^12^.

Thus, the objective of this study was to verify the physical-chemical stability of vancomycin hydrochloride in antimicrobial seal solutions with and without heparin sodium according to temperature and association time.

## Method

### Study design

An experimental research study, mimicking the clinical practice of antimicrobial seal administration in CICs.

### Study locus

Laboratory of Nursing Experiments (*Laboratório de Experimentos de Enfermagem*, LEEnf) of the São Paulo Nursing School, Federal University of São Paulo, São Paulo, SP, Brazil.

### Data collection period

Data collection took place between May and July 2018.

### Samples

Vancomycin hydrochloride solutions in physiological serum (PhS) and in association with heparin sodium, equivalent to those employed in the antimicrobial seal technique in central intravenous catheters.

### Sample definition

The sample consisted of 12 solutions, namely: three of vancomycin hydrochloride at 22°C, three of vancomycin hydrochloride at 37°C, three containing the association of antimicrobial and heparin sodium at 22°C, and three containing the association of antimicrobial and heparin sodium at 37°C. The solutions were prepared by a single researcher and the pH and concentration measurements occurred at the initial moment (T0) and at three (T3), eight (T8) and 24 hours (T24). Concentration checks were carried out in triplicates, resulting in 192 measurements (48 for pH and 144 for concentration). 

### Study variables

Simulating the clinical practice of antimicrobial seal administration, the experiments took place under controlled light and temperature conditions. The 37°C temperature aimed at mimicking body temperature. The drug concentrations were based on the Infectious Diseases Society of America (IDSA) Practice Guidelines^13^. Two different solutions were studied: vancomycin hydrochloride (5 mg/mL) and the association of the antimicrobial (5 mg/mL) with heparin sodium (100 I.U./mL). The conditions for the analysis programmed in the chromatograph were as follows: mobile phase (MP) in isocratic mode, flow at 1 mL/min; 30ºC column temperature, ultraviolet (UV) detector with wavelength at 220 nanometers (nm), injection of 20 microliters (μL) and analytical running time of 14 minutes (min.). 

### Instruments used for collecting the information

The pH of the solutions was studied in a digital bench top meter (Kasvi^®^ K39-2014B, Curitiba, Brazil) and the antimicrobial concentration by means of the high-efficiency liquid chromatography analytical methodology - HPLC (HPLC Modular Agilent Technologies^®^ - 1260 Infinity Series HPLC), using a 4.6 x 250 mm C18 reverse phase column and particle size of 5 micrometers (μm) (Thermo Scientific^®^ - ODS Hypersil, Massachussetts, United States of America, batch: 13359). For the daily cleaning procedure of the analytical column of the HPLC equipment, a solution composed of the mixture was established in the proportion of 80% methanol and 20% deionized water, for 20 minutes, with a 1 mL/min flow. Subsequently, the chromatographic parameters were programed. The conditions for the analysis programmed in the equipment were as follows: Mobile phase - MP (acetonitrile 8% and ionic pairing solution 92%) in isocratic mode, flow at 1 mL/min; 30ºC column temperature, UV detector with wavelength at 220 nm, injection of 20 μL and analytical running time of 14 minutes. Preparation of the solutions took place in a gas exhaust chapel (Sppencer^®^ SP1050-25, São Paulo, Brazil), with aseptic technique, with polypropylene syringes (3 mL and 10 mL) and sterile stainless steel needles (30.0 x 0.8 mm). Vancomycin hydrochloride (500 mg), heparin sodium (5,000 IU/mL) and PhS in a 100 mL flexible transparent polypropylene bag were used. The drugs, diluents and disposable materials used in the experiment were from the same manufacturing batch and respected shelf life. The experimental solutions were packed in amber glass bottles in order to mimic the absence of light to which the solutions are subjected when inside the catheters.

### Data collection

Validation of the analytical methodology was carried out by means of HPLC, prior to verifying the vancomycin hydrochloride concentration, based on the premises established in RE Resolution Number 166 of 2017 of the National Health Surveillance Agency (*Agência Nacional de Vigilância Sanitária*, ANVISA)^14^, in the ICH Q2(R1) guide of the 2005 International Conference on Harmonization^15^, and in the United States Pharmacopeia - USP 32^16^ for the selectivity, linearity, precision and accuracy parameters. After preparing the solutions, visual inspection and pH control were performed. To analyze the vancomycin hydrochloride concentrations, it was necessary to adapt to the chromatographic condition with adjustment of the drug sample for the linear working range of 0.1 mg/mL. 

### Data treatment and analysis

The results are presented based on the means of the triplicates of the pH checks and those of the concentrations in percentage and milligrams *per* milliliters, calculated taking into account the chromatographic peak area of vancomycin hydrochloride of each sample in the times established. Statistical analysis was performed using the multilevel linear regression model, analysis of variance (ANOVA) and multiple comparisons with Bonferroni correction. A 5% significance level was adopted for all the statistical analyses. The statistical analyses were performed using the SPSS 20.0 and Stata 12 programs.

### Ethical aspects

The study was submitted to the evaluation of Unifesp’s Research Ethics Committee (*Comitê de Ética em Pesquisa*, CEP) and approved under opinion No. 8942030117.

## Results

The results of the concentration and pH study were obtained by analyzing 12 vancomycin hydrochloride solutions and solutions containing the vancomycin hydrochloride-heparin sodium association. Table 1 shows the results of the concentration and pH study corresponding to the solutions according to time and temperature variations.

**Table 1:** Concentration and hydrogenionic potential of vancomycin hydrochloride solutions (n=6) and of the vancomycin hydrochloride-heparin sodium association (n=6) according to temperature and time. São Paulo, SP, Brazil, 2018

Temperature	Type of solution	Moment	Concentration (%) Mean ± SD (Min-Max)	Concentration (mg/mL) Mean ± SD (Min-Max)	Hydrogenioninc potential Mean ± SD (Min-Max)
22ºC	Vancomycin hydrochloride solution	T0	101.93 ± 1.66*	5.10 ± 0.08*	3.76 ± 0.05*
			(100.75-104.33)	(5.04-5.22)	(3.73-3.82)
		T3	101.45 ± 1.46	5.07 ± 0.07	3.73 ± 0.01*
			(99.54-103.58)	(4.98-5.18)	(3.73-3.74)
		T8	100.31 ± 0.87^†^	5.02 ± 0.04^†^	3.74 ± 0.01*
			(99.39-101.39)	(4.97-5.07)	(3.73-3.75)
		T24	102.50 ± 1.46*	5.12 ± 0.07*	3.59 ± 0.03^†^
			(100.52-104.44)	(5.03-5.22)	(3.56-3.62)
		p	<0.05	<0.05	<0.05
	Vancomycin hydrochloride and heparin sodium solution	T0	101.73 ± 3.81*	5.09 ± 0.19*	3.63 ± 0.03^†^
			(95.50-106.64)	(4.78-5.33)	(3.60-3.65)
		T3	99.01 ± 1.56^†^	4.95 ± 0.08^†^	3.59 ± 0.02^†^
			(97.49-101.24)	(4.87-5.06)	(3.58-3.61)
		T8	97.38 ± 1.20^†^	4.87 ± 0.06^†^	3.64 ± 0.04^†^
			(95.60-98.80)	(4.78-4.94)	(3.61-3.69)
		T24	96.34 ± 1.70^†^	4.82 ± 0.08^†^	4.00 ± 0.02*
			(94.36-99.44)	(4.72-4.97)	(3.99-4.02)
		p	<0.05	0.05	<0.05
37ºC	Vancomycin hydrochloride solution	T0	97.79 ± 0.69^†^	4.89 ± 0.03^†^	3.73 ± 0.02*
			(96.99-98.97)	(4.85-4.95)	(3.71-3.74)
		T3	101.96 ± 1.13*	5.10 ± 0.06*	3.69 ± 0.02^†^
			(100.25-102.92)	(5.01-5.15)	(3.68-3.71)
		T8	100.73 ± 1.69*	5.04 ± 0.08*	3.67 ± 0.01^†^
			(98.77-102.58)	(4.94-5.13)	(3.66-3.68)
		T24	98.35 ± 2.80^†^	4.92 ± 0.14^†^	3.68 ± 0.02^†^
			(95.41-101.83)	(4.77-5.09)	(3.66-3.69)
		p	<0.05	<0.05	0.05
	Vancomycin hydrochloride and heparin sodium solution	T0	94.91 ± 1.76^†^	4.75 ± 0.09^†^	3.80 ± 0.02
			(92.68-96.80)	(4.63-4.84)	(3.78-3.82)
		T3	95.12 ± 1.29^†^	4.76 ± 0.06^†^	3.75 ± 0.07
			(93.19-96.80)	(4.66-4.84)	(3.68-3.82)
		T8	95.05 ± 3.15^†^	4.75 ± 0.16^†^ (4.54-4.97)	3.75 ± 0.04
			(90.84-99.43)		(3.72-3.79)
		T24	96.94 ± 1.23*	4.85 ± 0.06*	3.78 ± 0.01
			(94.94-98.42)	(4.75-4.92)	(3.78-3.79)
		p	0.001	0.001	0.389

^*^Analysis of variance (ANOVA); ^†^Multiple comparisons Bonferroni correction; (^*^) and (^†^) present different means between the evaluation moments for each. The results are expressed through mean ± standard deviation

Table 1 shows a reduction in the concentration of vancomycin hydrochloride at 22°C at T0 and T8 with a subsequent increase in T24. Regarding the pH, there was a reduction over time.

Under the influence of the 37°C temperature, the vancomycin hydrochloride concentration was increased at the initial moment, as well as at three hours of preparation with a subsequent decrease at 24 hours (Table 1). Regarding pH, there was no significant variation in relation to the values found in the vancomycin hydrochloride solutions at 22°C with a reduction in the pH scale from the initial moment up to 24 hours, as shown in Table 1. 

In relation to the vancomycin hydrochloride-heparin sodium association solution at 22°C, regarding pH, a lower value was observed at three hours of preparation, and a higher value at 24 hours. Regarding the concentration, it presented a drop of around 5% in the antimicrobial values over time (Table 1). 

In the vancomycin hydrochloride with heparin sodium solutions, when submitted to 37ºC and with regard to pH, it is noted that they presented a higher value at T0, although with a smaller variation. As for the concentration, they presented a drop at the initial moment, reaching their maximum concentration 24 hours after preparation of the solutions (Table 1).

**Figure 1:**
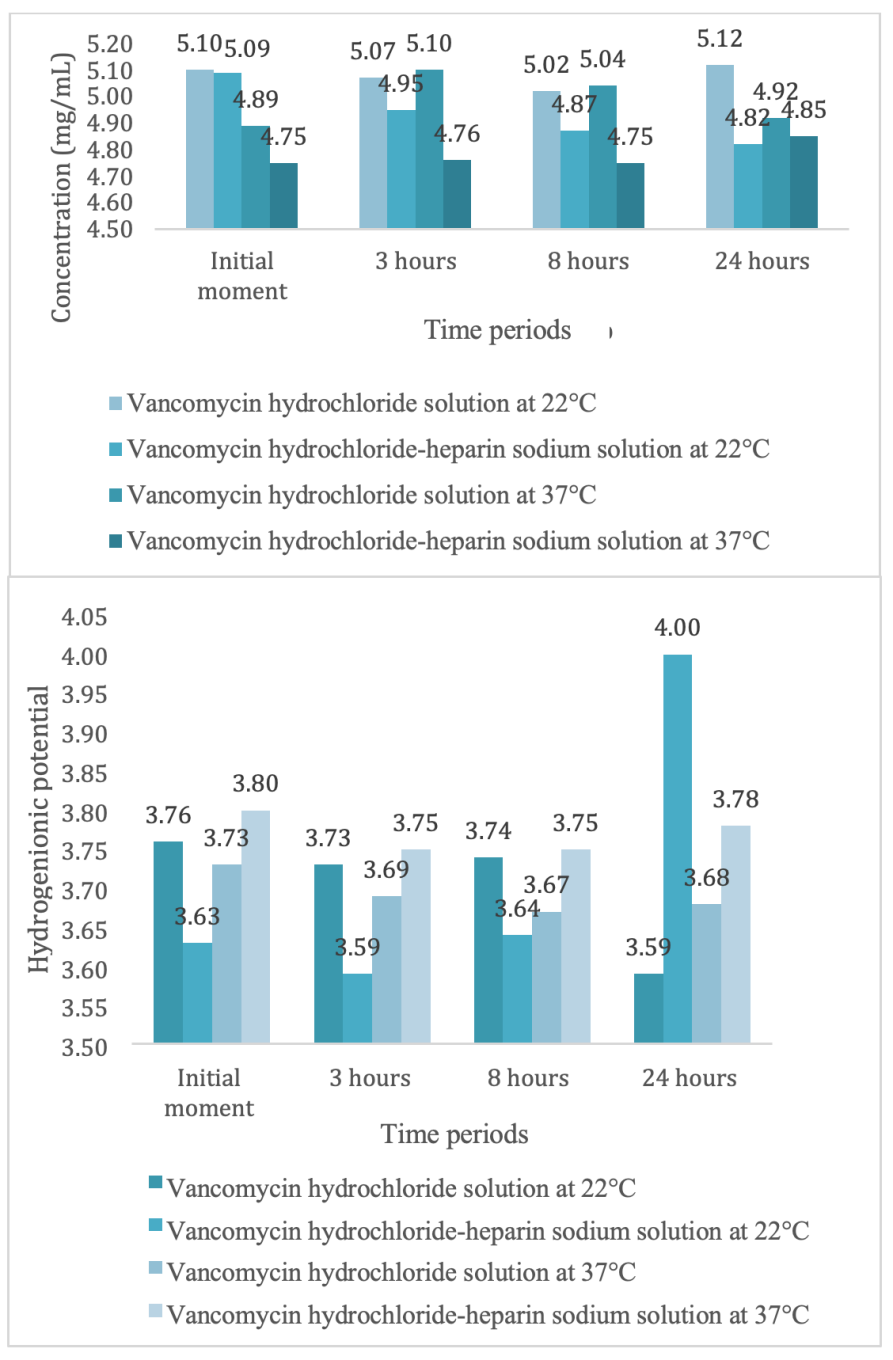
Mean values of concentration and hydrogenionic potential of the vancomycin hydrochloride solutions and of those of the vancomycin hydrochloride-heparin sodium association, according use time and exposure temperature. São Paulo, SP, Brazil, 2018

To graphically show the variation in concentration and pH obtained in the study, the results, according to the preparation time of the solutions, are presented in Figure 1.

*A = Concentration; ^†^ B = Hydrogenionic potential

Figure 1 shows that the 22°C temperature caused less variation in the concentrations; however, in the solutions containing the association of the antimicrobial with heparin sodium, it was possible to observe a reduction in concentration, even at 22°C. However, in the vancomycin hydrochloride-heparin sodium solution at 37°C, immediately after preparation, the concentration started with a value lower than the expected theoretical concentration (5 mg/mL).

Regarding the analysis of antimicrobial pH at both temperatures, it was observed that the values remained similar, except for 24 hours at 22°C, which proved to be more acidic (Table 1).

**Figure 2:**
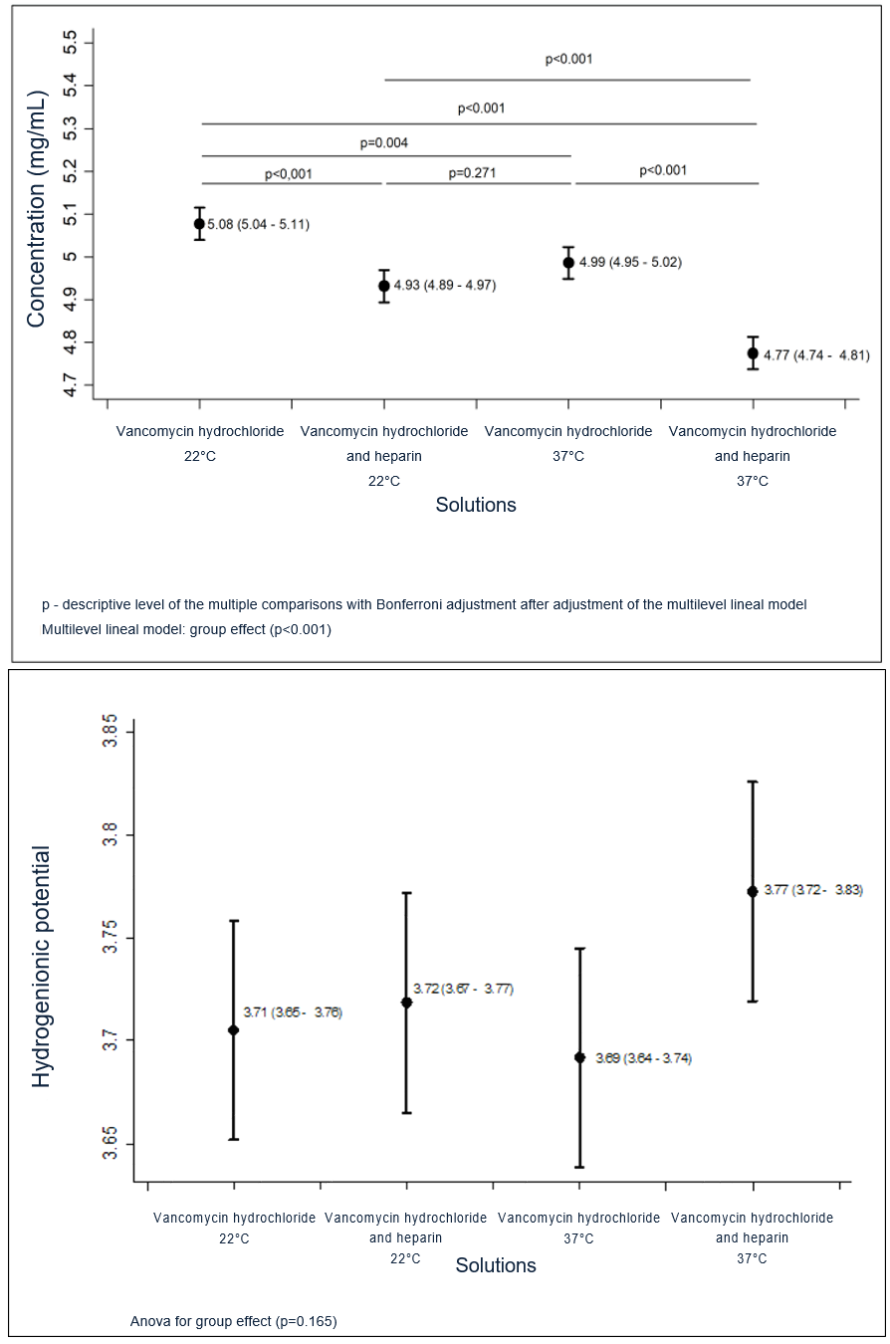
Concentration and hydrogenionic potential of the vancomycin hydrochloride solutions and of those containing the vancomycin hydrochloride-heparin sodium association, according to the influence of time and temperature on multiple paired comparisons (*a posteriori* Bonferroni test). São Paulo, SP, Brazil, 2018

Multiple comparisons were made with vancomycin hydrochloride solutions and solutions containing vancomycin hydrochloride in association with heparin sodium, at both temperatures and in the four time intervals established, as shown in Figure 2.

*A = Concentration; ^†^B = Hydrogenionic potential

The multiple mathematical comparisons performed in the study of the vancomycin hydrochloride solutions showed a statistically significant reduction in concentration of the antimicrobial in association with heparin sodium with pH elevation in the solutions submitted to 37ºC (Figure 2).

**Figure 3:**
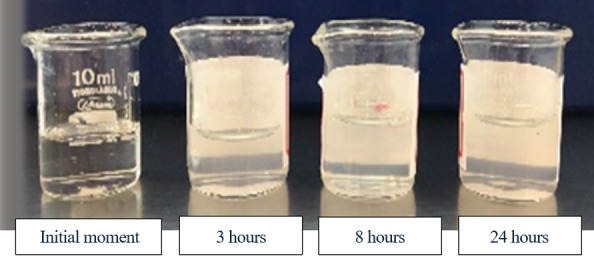
Vancomycin hydrochloride and heparin sodium solution at the different moment: initial; and after three, eight and 24 hours of use of the solutions at a temperature of 22°C; and with formation of a cloudy solution at three hours of preparation. São Paulo, SP, Brazil, 2018

Visual inspection of the solutions evidenced precipitate formation in the vancomycin hydrochloride-heparin sodium association at three hours, regardless of temperature (Figure 3).

## Discussion

The current study was carried out by means of a mimicking process of the clinical practice of the seal technique with antimicrobials in CICs, at temperatures of 22°C and 37°C. The concentrations of the vancomycin hydrochloride solutions at both temperatures were close to 100% (theoretical expected concentration of 5 mg/mL); they remained equivalent during the 24 hours after preparation, which corroborates the research regarding the evaluation of vancomycin hydrochloride concentration by the HPLC method that revealed pharmacological stability of the antimicrobial for up to 24 hours at room temperature^17^.

However, the association of vancomycin hydrochloride with heparin sodium resulted in precipitate formation at three hours at both temperatures, as well as in reduced antimicrobial concentration when at 37°C. 

It is known that the predominant profile of drug incompatibilities with potential risks for the patient is related to the class of antimicrobials, and the association between drugs may inactivate the active ingredient of the product or lead to toxicity, resulting in uncertainty of therapeutic efficacy^18^ ^-^ ^19^. Recognition of pharmacological incompatibilities makes it possible to avoid adverse situations, as well as the emergence of toxicity^20^ ^-^ ^21^.

To ensure the efficacy of vancomycin hydrochloride in decontamination of CICs, concentration of the drug should be maintained at a variation of less than 10% throughout the period in which the solution remains inside the intravenous device, also taking into account the materials of the CICs^22^. 

Analyzing the concentrations of the vancomycin hydrochloride solution at 22°C in the current experiment, in which the solutions remained packed in amber glass vials, it is noted that, despite the tenuous change in the values, the greatest variation occurred within periods of eight and 24 hours with a 2% increase in concentration of the antimicrobial at 24 hours of use of the solution (p>0.05), which decants the probability of the influence of time on stability of the drug.

Regarding pH, the vancomycin hydrochloride solution at 22ºC presented a reduction over time, accentuating the acid profile of the drug. In this situation, an extreme acidity value of all measurements was obtained; however, pH behavior over time was similar. Such values comprise the antimicrobial reference range from 2.5 to 5.5 according to the manufacturer^23^.

A study on drug stability states that extreme pH values can cause instability of the solutions and are considered key elements for physical-chemical compatibility of the solutions^24^.

Changes in the temperature to which the solutions are submitted can cause pH changes and consequent pharmacological instability^18^ ^,^ ^21^ ^,^ ^25^. However, in this research, regarding pH, the vancomycin hydrochloride solutions remained equivalent over time at 37°C. In addition, when comparing the vancomycin hydrochloride solutions at 22°C with the antimicrobial solutions at 37°C, a similar behavior is noted with reduced values over time and with a variation of less than 0.1 points in the pH scale.

When submitted to the 37°C temperature, the solution containing vancomycin hydrochloride in association with heparin sodium presented a higher pH mean value than the others found in the study, although with a reduction of 0.02 pH points from the initial moment to 24 hours after preparation. The greatest variation occurred in the solutions containing the association of the antimicrobial with heparin sodium at 22°C, with an increase of 0.37 pH points over time. Therefore, it can be suggested that in this experiment, high temperature did not affect the degree of concentration of hydrogen ions of the vancomycin hydrochloride solutions; however, there was a change in relation to the reduction of the antimicrobial concentration when in association with heparin sodium after 24 hours.

According to the American Pharmacopoeia^16^, the drug should comprise 90% to 110% of the labeled concentration. When comparing the concentrations of the solutions immediately after preparation, it was possible to observe a variation in the vancomycin hydrochloride concentration at the initial moment of preparation of the vials, although within the tolerance limit established. However, when evaluating the vancomycin hydrochloride concentration when in association with heparin sodium at 22°C, it was possible to observe a reduction of more than 5% in the values from the initial moment to 24 hours of use of the solutions. 

When establishing a comparison in this research between the solutions submitted to 22°C, it was possible to observe that the greatest variation in concentration occurred in the association with heparin sodium, with decreased antimicrobial concentration over time. When submitted to 37°C, the solution containing vancomycin hydrochloride and heparin sodium presented lower concentration values than the others found in the experiment. Regarding pH, the highest value obtained in the experiment was achieved in the vancomycin hydrochloride and heparin sodium solutions at 22°C at 24 hours, reaching a value of 4.0; therefore, 0.41 points higher than the value obtained in the antimicrobial solution at the same temperature and in the same period of time, a fact that suggests the interference of the anticoagulant in stability of vancomycin hydrochloride.

Solutions of vancomycin hydrochloride isolated or associated with heparin sodium were identified as the best alternatives for the treatment of CCRBSIs for Gram-positive microorganisms^26^ ^-^ ^27^. However, with regard to drug stability, the results were contradictory, especially for antimicrobial-heparin sodium association solutions since, although in one study^28^ vancomycin hydrochloride (5 mg/mL) was compatible with heparin sodium (2.5000 IU/mL), others, in turn, indicated that high concentrations of antimicrobial (10 mg/mL) can increase the risk for precipitation of the solutions, even with low doses of anticoagulant (100 IU/mL)^29^.

In this study, when at 22°C, there was a reduction in the vancomycin hydrochloride concentration after the association with heparin sodium in the evaluation of the solution after 3 hours of the 2.72% preparation. In a similar research study, a reduction in antimicrobial concentration was demonstrated in solutions containing vancomycin hydrochloride and heparin sodium inside CICs after 72 hours, suggesting, among other things, the interaction between the drugs as well as between the solutions and the intravenous device material^30^. 

A study conducted in a hospital found that most of the medication errors observed were related to drug incompatibility or to lack of evidence for administration associated to the drugs^31^. Drug incompatibility occurs when two or more drugs react or interact in a way that there is a change in the normal activity of one or more components, a fact that can derail clinical therapy, resulting in alteration of the active ingredient, precipitation or clouding of the solution and change in the color of the drug^20^ ^-^ ^21^ ^,^ ^32^.

A research on study vancomycin hydrochloride stability showed precipitate formation and turbidity when in association with heparin sodium from five minutes of solution preparation^29^. In the current study, changes were observed in the physical aspect of the solutions containing vancomycin hydrochloride in association with heparin sodium, evidenced by turbidity with precipitate formation at three hours at temperatures of 22ºC and 37ºC, suggesting interaction between the drugs. In addition, the formation of precipitate may result in obstruction of the intravenous device and/or risk of causing embolism in the patient, and the association between them is not recommended.

Feasibility of the occurrence of drug incompatibility and the scarcity of scientific evidence are difficulties found in nurses’ daily practice. It is to be remembered that performing drug associations without scientific evidence incurs in medication errors^33^ ^-^ ^34^. With regard to concentration of the drugs and solutions, it is possible that this may cause pharmacological instability and compromise effectiveness of the seal technique in CICs. Therefore, in order to better portray the clinical practice, it is suggested to investigate stability of vancomycin hydrochloride with a possible interference of different CIC materials, linked to the permanence time in the administration system as well as in the association between drugs and solutions.

The following stand out as study limitations: conduction of the experiments in glass vials, as well as the use of a single dosage of heparin sodium. Additionally, only one concentration of the antimicrobial (5 mg/mL) was performed. For future studies, it is suggested to investigate stability and concentration of vancomycin hydrochloride, when in association with heparin sodium under the possible interference of the material of intravenous devices and at a concentration of 10 mg/mL.

## Conclusion

There was a change in physical stability in the vancomycin hydrochloride (5 mg/mL) and heparin sodium (100 I.U./mL) solution at three hours of association with a reduction of less than 10% in antimicrobial concentration, evidencing chemical instability with drug degradation, although with pharmacological stability.

The vancomycin hydrochloride solutions maintained at 22°C presented the smallest variations in pH. However, temperature does not seem to be an environmental factor triggering considerable differences in the chemical behavior of the solutions studied.

Thus, this study evidenced that, regardless of temperature, the association of vancomycin hydrochloride with heparin sodium in solutions used in seals for CIC decontamination presented chemical instability and drug incompatibility. Despite maintenance of pharmacological stability, it is therefore recommended to adopt non-use of these compounds in association in the seal technique with an antimicrobial.
